# Shoulder Pain in Rheumatoid Arthritis: Ultrasound Findings and Clinical Correlations

**DOI:** 10.7759/cureus.84228

**Published:** 2025-05-16

**Authors:** Ganesh Kumar, Raj Kumar, Abhinav Singh, Deepak Kumar, Ranjeet Kumar, Sudhir Kumar

**Affiliations:** 1 Physical Medicine and Rehabilitation, Indira Gandhi Institute of Medical Sciences, Patna, IND; 2 Physical Medicine and Rehabilitation, Employees State Insurance Corporation (ESIC) Medical College and Hospital, Patna, IND; 3 Radiodiagnosis, Indira Gandhi Institute of Medical Sciences, Patna, IND

**Keywords:** high-resolution musculoskeletal ultrasound, rheumatoid arthritis, shoulder pain, shoulder pain and disability index, subacromial-subdeltoid bursitis

## Abstract

Aims

Shoulder pain is a common yet often overlooked manifestation of rheumatoid arthritis (RA). The prevalence and causes of shoulder pain in RA patients are not well-characterized, and imaging techniques, such as high-resolution musculoskeletal ultrasound (HRUS), have been underutilized in understanding its etiological profile. This study aims to evaluate the prevalence and explore underlying structural abnormalities (assessed via HRUS) and clinical characteristics of shoulder pain in patients with RA.

Materials and methods

An observational, cross-sectional study was conducted involving 208 RA patients. Patients were assessed using HRUS and clinical evaluations, including the Shoulder Pain and Disability Index (SPADI).

Statistical analysis

The study used descriptive statistics to summarize demographic and clinical data, calculated prevalence estimates for ultrasonography findings, and conducted group comparisons using one-way analysis of variance (ANOVA), Tukey's test, Kruskal-Wallis test, and Fisher's exact test.

Results

Shoulder pain was present in 105 out of 208 RA patients, yielding a prevalence of 50.48%. HRUS findings revealed that subacromial-subdeltoid (SASD) bursitis was the most common abnormality (50/105, 47.6%), followed by subcoracoid bursitis (35/105, 33.3%) and synovitis (16/105, 15.2%). Notably, 36 (34.3%) of patients exhibited no detectable HRUS abnormalities despite reporting pain. A significant association was found between seropositivity and shoulder pain, with 93.33% of patients with shoulder pain being seropositive.

Conclusion

Shoulder pain is prevalent among RA patients and is frequently associated with bursitis and synovitis, although it can occur without detectable HRUS abnormalities. Structural abnormalities correlate with increased pain severity and functional disability.

## Introduction

Shoulder pain is a common yet understudied manifestation of rheumatoid arthritis (RA), a chronic autoimmune disease characterized by synovial inflammation and progressive joint damage [1,2]. While RA predominantly affects small joints, larger joints, such as the shoulder, can also be involved, leading to significant pain, functional impairment, and reduced quality of life [3]. Despite its clinical relevance, the exact prevalence and underlying causes of shoulder pain in RA remain poorly characterized, with limited studies focusing on its etiological profile using high-resolution musculoskeletal ultrasound (HRUS) [3].

The shoulder joint’s complex anatomy and biomechanics make it susceptible to various pathological changes in RA, including synovitis, rotator cuff tears, bursitis, and bone erosions [2,4]. However, distinguishing between structural and non-structural causes of shoulder pain in RA patients remains a diagnostic challenge [5,6]. While some cases may be directly linked to synovial inflammation or tendon damage, others may arise from secondary factors such as neuropathic pain, referred pain, or systemic disease activity [7]. A comprehensive clinical evaluation using HRUS can help delineate these underlying abnormalities, guiding targeted therapeutic interventions.

Current literature suggests that shoulder involvement in RA may occur at any stage of the disease, but its prevalence and structural progression vary widely [8]. Early RA may present with subtle synovitis or effusions, whereas long-standing disease often leads to advanced degenerative changes, including rotator cuff tears and joint deformities [9]. Understanding these temporal patterns is crucial for early intervention, as delayed diagnosis may result in irreversible damage and increased disability [10]. Furthermore, the relationship between serological markers (such as rheumatoid factor (RF) and anti-cyclic citrullinated peptide (anti-CCP)) and shoulder pathology remains unclear, warranting further investigation.

Beyond structural abnormalities, systemic inflammation and metabolic factors may also likely contribute to shoulder pain in RA [11]. Additionally, functional disability-measured using tools like the Shoulder Pain and Disability Index (SPADI) may vary depending on the type and severity of HRUS-detected lesions, yet few studies have explored these correlations in RA patients [12,13].

Another critical yet often overlooked aspect is the subset of RA patients who experience shoulder pain without detectable structural abnormalities on imaging [14].

We hypothesize that shoulder pain in RA is highly prevalent and primarily driven by identifiable structural pathologies (e.g., synovitis, rotator cuff tears, or effusions) detectable on HRUS.

This study was conducted to evaluate the etiological profile of shoulder pain in RA patients by assessing its prevalence, underlying structural abnormalities (via HRUS), and clinical characteristics. By determining the prevalence of shoulder pain, characterizing associated HRUS findings, and correlating them with demographic and serological markers, this research seeks to enhance diagnostic precision and therapeutic decision-making. Additionally, the study compared disease duration and structural progression between early and late-stage shoulder involvement, offering insights into the natural history of RA-related shoulder pathology.

## Materials and methods

This research was conducted as a tertiary care, hospital-based, single-centered, cross-sectional study to evaluate the prevalence and characteristics of shoulder pain in RA patients using HRUS and clinical assessments. Approval was obtained from the institutional ethics committee of Indira Gandhi Institute of Medical Sciences, Patna, and written informed consent was secured from all participants.

The study was conducted in the Department of Physical Medicine and Rehabilitation (PMR) in a tertiary care hospital of eastern India. The study spanned approximately 6 months, during which 208 consecutive RA patients attending the PMR OPD were enrolled.

Sample size

Based on the estimated prevalence of shoulder pain in RA (ranging from 30% to 91% in the literature) [15,16], a sample size of 107 was initially calculated (assuming 50% prevalence, 95% confidence interval, 10% precision, and 10% attrition rate) using the formula:

\[ n = \frac{Z^2 \cdot P \cdot (1 - P)}{d^2} \]

The consecutive sampling method was used, and 208 patients were found to be eligible for our study. So, we enrolled all eligible patients to improve the power and generalizability of our study.

Eligibility criteria

Our study included consenting adult patients aged 18 years or older who were diagnosed with RA according to the 2010 American College of Rheumatology/European League Against Rheumatism (ACR/EULAR) criteria and presented with either unilateral or bilateral shoulder involvement [17]. Patients were excluded if they had functional shoulder pain or non-organic symptoms, psychiatric conditions affecting reliable history-taking, or shoulder pain attributed to referred causes, such as cervical radiculopathy, as confirmed by clinical examination.

Methodology

Patients fulfilling the inclusion and exclusion criteria were consecutively recruited for the study. After obtaining informed consent, demographic and clinical details were recorded. Each patient underwent a standardized HRUS of the affected shoulder(s) in the Department of Physical Medicine and Rehabilitation (PMR) at IGIMS, Patna. The HRUS was performed by a single trained physiatrist having more than 10 years of experience, following the technical guidelines of shoulder ultrasounds by the European Society of Skeletal Radiology (ESSR 2016) Guidelines for HRUS in Rheumatology [18]. HRUS was performed using the Fujifilm Sonosite M-Turbo L05323 system with a high-frequency 50 mm linear array transducer (6-15 MHz), following standardized scans per ESSR 2016 shoulder guidelines to evaluate glenohumeral effusion, synovitis and arthitis changes, bursitis (subacromial-subdeltoid (SASD), subcoracoid), supraspinatus tendon tears/tendinosis, infraspinatus tendon tears/tendinosis, subacapularis tendon tears/tendinosis, biceps tendon effusion, tendinitis, tear subluxation, etc. [18].

Patients were seated on a revolving stool to allow optimal access to the anterior, lateral, and posterior aspects of the shoulder. The long head of the biceps tendon was evaluated in both short- and long-axis planes with the arm in slight internal rotation and the elbow flexed at 90°. The subscapularis tendon was assessed during passive external and internal rotation, with the transducer swept along its length to ensure full visualization. The supraspinatus tendon was examined in both long- and short-axis orientations, using the intraarticular biceps tendon as a key landmark for proper transducer alignment. Dynamic assessment of subacromial impingement was performed by having the patient abduct the arm in internal rotation while imaging the supraspinatus and subacromial-subdeltoid bursa [18].

Posterior structures, including the infraspinatus and teres minor tendons, were evaluated with the transducer placed over the posterior glenohumeral joint, using the scapular spine as an anatomical reference. The posterior labrum-capsular complex and spinoglenoid notch were assessed for the exclusion of effusion or paralabral cysts, with adjustments made to imaging depth as needed. The acromioclavicular joint was examined in the coronal plane, with anterior and posterior sweeps to assess ligament integrity and detect and exclude any os acromiale. Throughout the examination, care was taken to avoid and minimize anisotropy by adjusting transducer tilt and orientation. This systematic approach ensured a comprehensive evaluation of shoulder anatomy and dynamic function [18].

The severity of shoulder pain and functional disability was evaluated using the Shoulder Pain and Disability Index (SPADI), a well-validated, open-access, patient-reported outcome measure specifically designed for shoulder pathologies [19]. The SPADI consists of 13 items divided into two subscales: the Pain Subscale (5 items), which assesses the intensity of pain during various activities such as resting, reaching overhead, and lying on the affected side, and the Disability Subscale (8 items), which evaluates functional limitations in daily tasks like washing hair, carrying heavy objects, and dressing [19].

Each item is scored on a visual analog scale ranging from 0 (no pain/no difficulty) to 10 (worst imaginable pain/unable to perform the activity), with total scores converted to a percentage (0-100%). Higher scores indicate greater pain severity and functional impairment, providing a quantitative measure of shoulder-related disability. The SPADI has demonstrated high reliability (test-retest reliability >0.90) and validity in clinical and research settings, making it a robust tool for assessing treatment outcomes in shoulder disorders [19].

To ensure accuracy, participants were provided with standardized instructions and supervised while completing the questionnaire. Baseline and follow-up SPADI scores were compared to evaluate changes in pain and function over time. This method allowed for a comprehensive, patient-centered assessment of shoulder dysfunction in alignment with established clinical research protocols.

Data collection included demographics such as age, gender, and disease duration, clinical variables, including SPADI scores (pain, disability, total), laterality of shoulder involvement, and comorbidities, as well as HRUS findings recorded as categorical variables indicating the presence or absence of specific pathologies.

Laboratory parameters encompassed inflammatory markers (hemoglobin, total leukocyte count, neutrophils, lymphocytes, platelets, erythrocyte sedimentation rate), metabolic markers (random blood sugar, thyroid-stimulating hormone, vitamin D levels), liver function tests (serum glutamic-oxaloacetic transaminase, serum glutamic-pyruvic transaminase), and serological status (rheumatoid factor and anti-cyclic citrullinated peptide) to classify patients as seropositive (RF/anti-CCP+) or seronegative (RF/anti-CCP−) for rheumatoid arthritis. While all lab values were collected, only serological status was considered for analysis in this article.

Statistical analysis

Descriptive statistics, including mean ± standard deviation, median with interquartile range, and percentages, were used to summarize demographic and clinical data. Prevalence estimates for ultrasonography (HRUS) findings were calculated with Wald 95% confidence intervals. Group comparisons were conducted using one-way analysis of ANOVA, followed by Tukey’s test for age, the Kruskal-Wallis test for non-parametric variables, such as disease duration and SPADI scores, and Fisher’s exact test for categorical data like gender distribution. A p-value of <0.05 was considered statistically significant.

## Results

Our study enrolled 208 RA patients, of whom 105 experienced shoulder pain, resulting in a prevalence of 50.48% (95% CI: 43.69-57.27). To further investigate, HRUS was performed on patients with shoulder pain. We found no statistical correlation between shoulder pain and other demographic variables.

Table 1 presents the distribution of HRUS findings among 105 patients with shoulder pain, revealing that subacromial-subdeltoid (SASD) bursitis was the most common abnormality (50/105, 47.6%, 95% CI: 38.0-57.3%), followed by subcoracoid bursitis (35/105, 33.3%, 95% CI: 24.3-42.4%) and cases with no detectable HRUS findings (36/105, 34.3%, 95% CI: 25.2-43.4%). Less frequent findings included synovitis (16/105, 15.2%), biceps tendon effusion (15/105, 14.3%), glenohumeral (GH) effusion (13/105, 12.4%), arthritis (14/105, 13.3%), and erosion (3/105, 2.9%), while tear generation/tendinosis was rare (2/105, 1.9%). The 95% confidence intervals indicate variability in prevalence estimates, with SASD bursitis showing the highest diagnostic yield.

**Table 1:** Distribution of patients with shoulder pain with respect to HRUS findings SASD: Subacromial-Subdeltoid; GH: Gleno-Humeral; HRUS: High-Resolution Musculoskeletal Ultrasound; CI: Confidence Interval

HRUS Findings	Number of Patients	% (n=105)	95% CI of %
Synovitis	16	15.2%	(8.4% – 22.1%)
SASD	50	47.6%	(38.0% – 57.3%)
Subcoracoid Bursitis	35	33.3%	(24.3% – 42.4%)
Biceps Tendon Effusion	15	14.3%	(7.6% – 21.0%)
GH Effusion	13	12.4%	(6.0% – 18.8%)
Tear Generation/Tendinosis	2	1.9%	(0.0% – 4.6%)
Arthritis	14	13.3%	(6.8% – 19.9%)
Erosion	3	2.9%	(0.0% – 6.1%)
No HRUS Findings	36	34.3%	(25.2% – 43.4%)

Table 2 compares baseline demographic and clinical characteristics between patients with different HRUS findings for shoulder pain, revealing that age (mean ± SD) varied across groups, with arthritis patients being the oldest (53.93 ± 12.96 years) and erosion cases the youngest (38.67 ± 15.82 years), though overall age differences were not statistically significant (p=0.1073, one-way ANOVA) except for a notable difference between SASD bursitis and arthritis (p<0.05, Tukey’s test). Males constituted 19.05% (20/105) of the total cohort, with no significant gender differences between groups (p=0.1913, Fisher’s exact test). Disease duration (median (IQR)) differed significantly (p=0.0218, Kruskal-Wallis test), with arthritis patients reporting the longest duration (10 years) and those with no HRUS findings the shortest (2 months), suggesting that structural abnormalities like arthritis may correlate with prolonged symptoms, whereas normal HRUS findings may indicate earlier or less severe pathology.

**Table 2:** Comparison of baseline demographic and clinical characteristics between various HRUS findings *One-Way ANOVA, **Tukey’s Multiple Comparison Test, ***Kruskal-Wallis Test, # Fisher’s Exact Test (With HRUS Findings vs No HRUS Findings) SD: Standard Deviation; IQR: Inter-Quartile Range; SASD: Subacromial-Subdeltoid; GH: Gleno-Humeral; HRUS: High-Resolution Musculoskeletal Ultrasound

HRUS Findings	Age in Years, Mean ± SD	Number of Males (%)	Number of Females (%)	Duration of Disease, Median (IQR)
Synovitis (n=16)	43.94 ± 12.57	3 (18.75)	13 (81.25)	4.5 (2-7.5)
SASD (n=50)	41.8 ± 10.57	12 (24)	38 (76)	3 (1.5-5.25)
Subcoracoid Bursitis (n=35)	45.4 ± 12.78	6 (17.14)	29 (82.86)	4 (2-8)
Biceps Tendon Effusion (n=15)	41.33 ± 10.53	4 (26.67)	11 (73.33)	4 (2-6)
GH Effusion (n=13)	46 ± 14.12	1 (7.69)	12 (92.31)	5 (2-10)
Tear Generation/Tendinosis (n=2)	54.5 ± 24.75	1 (50)	1 (50)	6.5 (3-10)
Arthritis (n=14)	53.93 ± 12.96	3 (21.43)	11 (78.57)	10 (1.375-10.5)
Erosion (n=3)	38.67 ± 15.82	1 (33.33)	2 (66.67)	3 (0.5-3)
No HRUS Findings (n=36)	45.72 ± 14.83	4 (11.11)	32 (88.89)	2 (1-4.75)
Total (n=105)	44.82 ± 13.52	20 (19.05)	85 (80.95)	4.5 (2-7.5)
P-Value	0.1073* (P<0.05** for SASD vs Arthritis)	0.1913#		0.0218***

Patients with tear generation/tendinosis (n=2) and erosion (n=3) exhibited the highest median pain (39% and 40%, respectively) and total SPADI scores (35.77 and 36.15), suggesting greater shoulder dysfunction, while those with synovitis (n=16) had the lowest median pain (33%) and total SPADI score (29.62). SASD (n=50) and biceps tendon effusion (n=15) also showed moderately high pain and disability scores. Notably, patients with no HRUS abnormalities (n=36) had median scores (pain: 35%, total SPADI: 32.31) close to the overall cohort median (pain: 34%, total SPADI: 31.54), indicating that clinical symptoms may persist even without detectable structural pathology. The variability in IQRs (e.g., wide ranges in erosion and arthritis) highlights heterogeneity in symptom severity within subgroups. Overall, structural abnormalities like tears and effusions were associated with worse SPADI scores, though pain and disability were present across all groups, including those without imaging findings (Table 3).

**Table 3:** Comparison of SPADI scores between patients with various HRUS findings SPADI: Shoulder Pain and Disability Index; SASD: Subacromial-Subdeltoid; GH: Gleno-Humeral; HRUS: High-Resolution Musculoskeletal Ultrasound; IQR: Inter-Quartile Range

HRUS Findings	Pain Scale in %, Median (IQR)	Disability Scale in %, Median (IQR)	Total SPADI, Median (IQR)
Synovitis (n=16)	33 (28-39.5)	28.13 (25-31.88)	29.62 (25.77-35.38)
SASD (n=50)	36 (29.5-42)	30 (25-35)	31.92 (26.54-37.12)
Subcoracoid Bursitis (n=35)	34 (28-42)	30 (23.75-33.75)	30.77 (23.85-36.15)
Biceps Tendon Effusion (n=15)	38 (30-44)	31.25 (27.5-36.25)	34.62 (29.23-39.23)
GH Effusion (n=13)	36 (30-41)	28.75 (26.88-35)	31.54 (28.85-36.54)
Tear Generation/Tendinosis (n=2)	39 (34-44)	33.75 (31.25-36.25)	35.77 (32.31-39.23)
Arthritis (n=14)	36 (26.5-40.5)	31.88 (24.38-35.31)	33.85 (25-37.12)
Erosion (n=3)	40 (34-46)	33.75 (30-36.25)	36.15 (31.54-40)
No HRUS Findings (n=36)	35 (28.5-39.5)	30.63 (26.56-32.5)	32.31 (27.31-35.96)
Total (n=105)	34.00 (30.0-40.0)	30.0 (26.25-33.75)	31.54 (26.92-36.15)
P-Value (Krushkal Wallis Test)	0.7782	0.6168	0.6483

**Figure 1:**
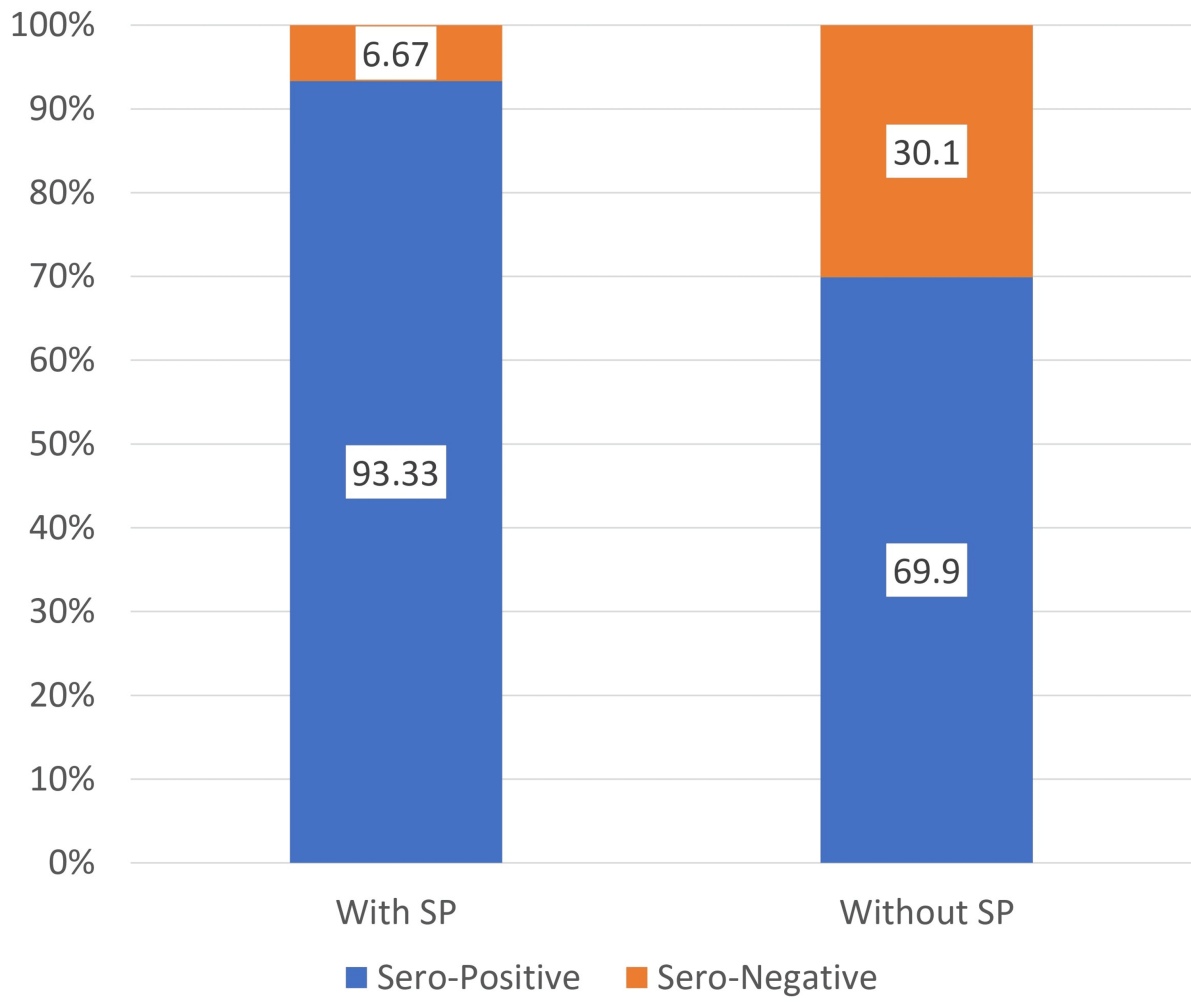
Comparison of sero-positive cases between patients with shoulder pain and without shoulder pain SP: Shoulder Pain

Figure 1 compares seropositivity rates between RA patients with and without shoulder pain, revealing a highly significant difference (p<0.0001). Among 105 patients with SP, 98 (93.33%) were seropositive, whereas only 72 (69.90%) of 103 patients without shoulder pain were seropositive. Conversely, seronegative cases were substantially more common in patients without shoulder pain (31/103, 30.10%) compared to those with shoulder pain (7/105, 6.67%). These findings suggest that seropositive RA is significantly associated with shoulder pain. No significant association between HRUS findings and serological status was found.

## Discussion

Shoulder involvement in RA is a well-documented yet frequently underrecognized complication, often masked by overlapping symptoms of degenerative or mechanical shoulder disorders. Ultrasonography (HRUS) has revolutionized the detection of RA-related shoulder pathology by enabling non-invasive visualization of synovitis, bursitis, tendon damage, and erosions-key features of disease progression. Previous research has established that HRUS can identify subclinical shoulder involvement even in asymptomatic RA patients (Elbinoune et al., 2016; Sakellariou et al., 2013) [20,21], with synovitis, bursitis, and effusions being the most common findings (Stegbauer et al., 2008; Leblebicier et al., 2021) [2,22]. Moreover, seropositive RA patients exhibit a higher prevalence of shoulder pathology (Stegbauer et al., 2008) [22], and HRUS has been shown to outperform clinical examination in diagnosing rotator cuff tears and inflammatory lesions (Naredo et al., 2002) [23]. These findings underscore the importance of imaging in the comprehensive management of RA.

Our study builds upon this foundation, revealing several clinically significant insights. Among 208 RA patients, shoulder pain was present in 105 (50.48%), aligning with prior reports (Sakellariou et al., 2013) [21]. Ultrasonographic evaluation of these patients identified subacromial-subdeltoid (SASD) bursitis (50/105, 47.6%) and subcoracoid bursitis (35/105, 33.3%) as the most prevalent abnormalities, consistent with earlier studies that reported subacromial bursitis in 35-37.8% of cases (Stegbauer et al., 2008; Elbinoune et al., 2016) [20, 22]. Notably, 36 (34.3%) of patients had no detectable HRUS abnormalities despite reporting pain, a finding that contrasts with studies where nearly all patients exhibited pathological changes (Leblebicier et al., 2021) [2]. This discrepancy suggests that pain in RA may sometimes arise from non-structural causes, such as early inflammation not yet visible on HRUS, neuropathic mechanisms, or extra-articular manifestations, warranting further investigation [7].

One of the most striking observations was the strong association between seropositivity and shoulder pain, reinforcing the link between autoantibody positivity and musculoskeletal involvement (Stegbauer et al., 2008) [22]. Additionally, patients with arthritis had the longest disease duration (median: 10 months), supporting the notion that chronic RA leads to cumulative structural damage (Sakellariou et al., 2013) [21].

When compared to prior research, our findings both corroborate and expand existing knowledge. For instance, the predominance of bursitis in our cohort mirrors reports by Stegbauer et al. (2008) and Elbinoune et al. (2016), confirming its role as a hallmark of RA shoulder involvement [20,22]. However, the absence of HRUS abnormalities in over a third of symptomatic patients diverges from studies where pathological findings were nearly universal (Leblebicier et al., 2021), suggesting that clinical symptoms may precede detectable structural changes [2]. This discrepancy underscores the need for complementary imaging modalities, such as MRI, in early disease stages.

Similarly, the strong association between seropositivity and shoulder pain reinforces the findings of Stegbauer et al. (2008), who reported glenohumeral synovitis in 68% of serologically active RA patients [22]. Our data also highlight the progressive nature of shoulder damage, with arthritis patients exhibiting longer disease duration - a trend consistent with Sakellariou et al. (2013), who linked inflammatory shoulder involvement to higher disease activity and chronicity [21].

The clinical implications of these findings are profound. Routine shoulder HRUS may be integrated into RA management protocols, particularly for seropositive patients, to facilitate early detection and intervention. The high prevalence of bursitis and synovitis supports the use of targeted therapies, such as corticosteroid injections or biologic agents, to mitigate pain and prevent irreversible damage. Conversely, pain without HRUS abnormalities necessitates a broader diagnostic approach, potentially incorporating neurological and detailed musculoskeletal evaluation or advanced imaging to identify alternative etiologies.

Limitations 

Due to the cross-sectional design, the assessment of longitudinal changes in shoulder pathology and pain progression in RA patients was not done. The absence of advanced imaging, such as MRI or power Doppler, may have led to under-detection of subtle inflammatory changes. The relatively small sample size may not be sufficient to detect subtle specific findings, particularly for erosion or tears, in detail. Larger studies will be needed to further investigate these aspects.

Future research should focus on longitudinal HRUS studies to track the evolution of shoulder pathology and the role of power Doppler and MRI in early disease. For detailed or further details about the percentage of specific findings, like erosion, or structural abnormalities like tear/tendinosis, etc., studies with a larger sample size should be conducted. Additionally, investigating the mechanisms of pain in HRUS-negative patients could uncover novel therapeutic targets. By addressing these gaps, we can refine diagnostic strategies and improve outcomes for RA patients with shoulder involvement, ultimately preserving function and quality of life.

## Conclusions

Shoulder pain is highly prevalent (nearly 50%) among patients with rheumatoid arthritis (RA) and is frequently associated with bursitis and synovitis (in one-third to half of cases), although it can also occur without detectable ultrasonographic (HRUS) abnormalities. Structural abnormalities, including tears and effusions, are linked to increased pain severity and greater functional disability. Additionally, seropositive RA shows a strong correlation with shoulder pain, with elevated inflammatory markers observed in patients presenting with effusions and synovitis. These findings highlight the importance of a comprehensive clinical evaluation using HRUS to ensure effective management of shoulder pain in RA patients. Our study underscores the critical role of HRUS in RA shoulder assessment, while also highlighting its limitations in early or non-structural disease. By integrating imaging with clinical and serological data, clinicians can deliver more precise and personalized care, aligning with the evolving paradigm of RA management. To strengthen this evidence, there is also a need for future prospective, interventional studies.
